# What can your microbiota tell you about your health?

**DOI:** 10.1590/1806-9282.20240862

**Published:** 2024-11-22

**Authors:** Ozgenur Hacioglu, Zeynep Ozoral

**Affiliations:** 1 Kirklareli University, Faculty of Health Sciences, Department of Midwifery – Kırklareli, Turkey.

**Keywords:** Microbiota, Education, Awareness

## Abstract

**OBJECTIVE:**

As healthy microbiota lays the foundation, it is crucial for midwives, who play an important role and responsibility in providing necessary recommendations for strengthening the connection between the mother and the child, to understand the basic structures and functions of various microbial communities and to be knowledgeable about microbiota and health. Therefore, this study aimed to provide microbiota education to midwifery students to explain the importance of microbiota for health, demonstrate its relationship with newborn health, women's health, and diseases, and evaluate the students’ awareness of microbiota at the end of the education.

**METHODS:**

The education provided within the scope of the research was conducted face-to-face over 4 weeks, with 2 h of education per week, totaling 8 h. The students’ level of knowledge about microbiota was assessed before and 3 months after the education using the microbiota knowledge test, and statistical analysis was conducted.

**RESULTS:**

The education provided within the scope of the research was conducted face-to-face over 4 weeks, with 2 h of education per week, totaling 8 h. The students’ level of knowledge about microbiota was assessed before and 3 months after the education using the microbiota knowledge test, and statistical analysis was conducted.

**CONCLUSIONS:**

Through peer education, areas of confusion experienced by students regarding microbiota were gamified, resolved through mutual knowledge exchange, and awareness of microbiota was raised.

## INTRODUCTION

The term "microbiota" refers to all microorganisms found in humans and is known to play an important role in maintaining the balance between health and disease in the human body^1^. Factors such as physiological status, environmental and cultural factors, nutrition, antibiotic use, and hygiene conditions have been indicated to be influential in the development of microbiota^2^. Recent studies on microbiota and health have reported significant roles of human microbiota in maintaining health. Microbiota has positive effects on health. In recent years, various studies have further elucidated the mechanisms by which microbiota benefits women's health. In particular, maintaining vaginal health and consuming probiotics that regulate microbiota contribute to improved birth-related outcomes. This process also has an indirect positive impact on newborn health^3-7^.

Due to the foundational role of a healthy microbiota, it is crucial for midwives, who have an important role and responsibility in providing necessary recommendations for strengthening the connection between the mother and the child, to understand the basic structures and functions of various microbial communities and to be knowledgeable about microbiota and health. While there is a scarcity of studies evaluating the knowledge levels of midwifery students on microbiota, in a study evaluating the knowledge levels of midwifery students on intestinal microbiota, which is known as the second metabolic organ, researchers reported the need to increase the knowledge levels of midwifery department students on intestinal microbiota^8^. In another study involving midwives who are actively engaged in professional life, it was evaluated that the knowledge level and awareness of midwives and nurses working in obstetrics clinics need to be increased regarding microbiota^9^.

In various studies, attempts have been made to measure individuals’ levels of awareness regarding microbiota, and some health professionals have been provided with education, seminars, and congresses on the subject^10^. However, it is observed that almost all of these efforts are at the postgraduate and professional education levels. Increasing educational interventions aimed at enhancing microbiota awareness among undergraduate students on the path to becoming health professionals will serve similar purposes. In this regard, knowledge about microbiota among midwifery candidates can contribute to the protection and development of maternal and child health. This study aims to provide microbiota education to midwifery students and evaluate their achievements in this area. The training was conducted using the peer education model, which is an educational approach that benefits students’ knowledge and skills. In addition, as far as we have seen in the literature review, it was determined that there is no study aiming to provide microbiota awareness education to midwifery students using the peer education model. Therefore, it is believed that this study could serve as a model for future educational research on microbiota.

## METHODS

### Data collection tools

The research was conducted semi-experimentally. In the study, a participant recognition questionnaire and a microbiota knowledge test were used as data collection tools. The microbiota knowledge test (the test form to be used for both pre-test and post-test) consisted of 20 questions related to the topics covered in the education, and participants were asked to answer them as "true" or "false." Participants received 5 points for each correct answer, with no points deducted for incorrect answers. A participant who answered all questions correctly received a total of 100 points. Participants completed the microbiota knowledge form as both pre-test and post-test, and their pre-test and post-test scores were compared. The education was conducted for 4 weeks, totaling 8 h of education.

### Evaluation of data

The data obtained from the research were analyzed using the Statistical Package for the Social Sciences 22 (SPSS 22.0) program. Descriptive statistical methods (frequency, percentage, minimum–maximum values, median, mean, and standard deviation) were utilized in evaluating the data, and the normal distribution of the data was assessed using the Kolmogorov-Smirnov test. For data not showing normal distribution, the Wilcoxon test was employed, while the dependent samples t-test was used for data showing normal distribution. Results were evaluated at a significance level of p<0.05 and p<0.001 with a 95% confidence interval.

### Ethical aspect

Ethics committee approval to conduct the present study was obtained from the Kirklareli University Scientific Research and Publication Ethics Committee (Number: E-35523585-302.99-102919, dated November 7, 2023). The purpose and method of the study were explained to the participants before its commencement. All participants gave written informed consent before inclusion. All practices were carried out in accordance with the 1964 Helsinki Declaration.

## RESULTS

### Evaluation of participant information form

A total of 63 (n=63) students participated in the education. All participants were second-year students in the Midwifery Department and were female. The mean age of the participants was 20.2±1.10 years, with the oldest being 25 and the youngest 19. When the consumption/use of probiotic products by participants was examined, it was determined that 25.4% (16/63) bought probiotic products, while 74.6% (47/63) did not consume/use them for various reasons (Table 1). Evaluation of participants’ preferred sources of information on microbiota revealed that the internet was the most commonly cited source (68%, n=43/63), followed by healthcare professionals (32%, n=20/63). When examining participants’ attendance rates at educational programs such as seminars or congresses on microbiota, it was found that 59% (n=37/63) answered "no," while 41% (n=26/63) answered "yes."

**Table 1:** The consumption/use of probiotic products by participants.

Do you consume/use probiotic products?	Yes 25.4% (n: 16/63)
Orally by capsule	9
Orally with powder	2
Vaginal with suppository	0
Nasal with spray	0
Apply the cream to the skin surface	5
	**No 74.6% (n: 47/63)**
I find the use of probiotics unnecessary	2
I don't know about its importance	16
I don't find it delicious	10
I cannot afford to buy probiotic products	6
Other	13
Total number of participants	100% (n: 63)

### Pre-test application

It was determined that the average score of the students in the microbiota knowledge test pre-test application was 77.5/100.

### Post-test application

Responses to statements in the microbiota test by students were evaluated based on their post-education answers. All students, 100% (63/63), correctly answered the statement "The entirety of microorganisms found in humans is called microbiome, and its genome is called microbiota" as "false." Similarly, 100% (63/63) of students correctly answered the statement "Microbiota varies depending on numerous endogenous and exogenous factors." Regarding the statement "Microbiota has metabolic, trophic, and protective functions," 92.1% (58/63) of students provided correct answers. The statement "Antibiotic use has no adverse effect on gut microbiota" was correctly answered as "false" by 96.8% (61/63) of students. Regarding the association between dysbiosis in the microbiota and intestinal cancer, 85.7% (54/63) of students correctly answered the statement. Additionally, 98.4% (62/63) of students correctly answered the statement, "Prebiotics are undigested food compounds that support beneficial bacteria in the flora." Similarly, 98.4% (62/63) of students correctly answered the statement "Probiotics are live microorganisms that positively impact human health when consumed in adequate amounts." The statement "Probiotics should not be used regularly" was correctly evaluated as "false" by 96.8% (61/63) of students. Furthermore, 100% (63/63) of students correctly answered the statement "Probiotics are not found in fermented dairy products." The statement "*Candida* is the most commonly encountered type of fungus in the oral cavity" was correctly answered by 100% (63/63) of students. Regarding vaginal microbiota, 96.8% (61/63) of students correctly answered the statement "Estrogen plays a role in shaping vaginal microbiota" as "false." Similarly, 58.7% (37/63) of students correctly answered the statement "Lactobacilli raise vaginal pH by producing lactic acid" as "false." Additionally, 90.5% (57/63) of students correctly answered the statement "*Lactobacillus acidophilus* is one of the Döderlein bacilli found in the vagina." The statement "An increase in *Gardnerella* bacteria count contributes to the disruption of healthy vaginal microbiota" was correctly answered by 98.4% (62/63) of students. Regarding "Fecal Microbiota Transplantation," 96.8% (61/63) correctly identified the statement as "the process of transferring stool from a healthy donor to a patient's gastrointestinal tract." Similarly, 98.4% (62/63) of students correctly answered the statement "The uterus, fallopian tubes, and ovaries have a microbial flora" as "true." The statement "The diversity of infant gut microbiota is not determined by the method of delivery" was correctly evaluated as "false" by 88.9% (56/63) of students. Additionally, 96.8% (61/63) of students correctly answered the statement "The initial colonization of newborns’ skin occurs through maternal vaginal microbiota during vaginal delivery." Furthermore, 84.1% (53/63) of students correctly identified the statement, "Formula milk is not the same as maternal milk microbiota." Finally, 100% (63/63) of students correctly answered the statement, "The beneficial flora bacterium in breast milk is Bifidobacterium."

In the post-test application of the microbiota knowledge test, the average score obtained by participants was 93.3/100. It was determined that among the 63 participants attending the training, 8 (12.7%) had unchanged pre-test and post-test scores. However, 55 (87.3%) participants showed a significant increase in their post-test scores. The percentage increase in knowledge at the end of the training for participants was 20.5%. When comparing participants’ pre-test (77.5/100) and post-test (93.3/100) knowledge scores, a statistically significant difference was observed (p<0.001) (Table 2). Upon examination of the responses provided by participants in the training satisfaction evaluation form, it was found that the participants unanimously agreed that the microbiota training program was adequate, with 100% agreement (n=63). Regarding questions about the adequacy of the materials used in the training, the duration of the training, and the content of the training, participants reported that the training materials were adequate by 88.9% (56/63), the duration of the training was adequate by 90.5% (57/63), and the content of the training was adequate by 87.3% (55/63).

**Table 2:** Comparison of participants’ knowledge scores before and after training.

Variables	Pre-test (Mean±SD)	Post-test (Mean±SD)	p*
Knowledge scores	77.5±11.77	93.3±6.72	<0.001

Data are represented as mean±SD. Mean: arithmetic mean, SD: standard deviation.

* Paired samples t-test.

## DISCUSSION

The microbiota encompasses all microorganisms present in humans, making it impossible to overlook its profound role in health^1^. In the literature, it is acknowledged that participants’ levels of microbiota knowledge may vary according to education level, age, and areas of expertise^8-15^. Among different education levels, university students’ awareness of probiotics varies depending on their fields of specialization, with most students being able to associate probiotic consumption with health, but exhibiting deficiencies in knowledge regarding microbiota concepts and particularly their relationship with diseases^13^. Studies in the literature on individuals’ microbiota knowledge mostly focus on probiotics and their consumption^11^. In this study, 63 second-year midwifery students participated, all of whom were female with an average age of 20.2 years. It is generally known that individuals mostly learn about probiotics through media communication channels, especially social media^10-12^. Similarly, when evaluating the preferred sources of information on microbiota among the participating students, it was found that the internet was the most common source (68%), followed by healthcare professionals (32%).

In studies conducted with university students in various countries, it has been reported that questions asking for explanations of microbiota-related concepts were preferably left blank, some were answered incorrectly, or students recorded their general knowledge on the topic as insufficient in the data form^11,12^. Consistent with the literature, we can conclude that the inclusion of the microbiology course in the midwifery department curriculum may have contributed to the high pre-test scores of second-year students.

In a study, the knowledge levels and consumption statuses of probiotic products among 247 nursing and midwifery students were aimed to be determined. It was indicated that the majority of the participating students were familiar with the concept of probiotics and responded to questions related to probiotic source products. The research also identified a relationship between knowing the concept of probiotics and gender and department. As a result of the study, it is considered necessary to inform nursing and midwifery students about the concept of probiotics^15^. In this study, it was determined that the average score of the students in the microbiota knowledge test in the pre-test application was 77.5/100. The familiarity of the participants with the topic could be explained by their connection to microbiota, as they had seen the connection in the curriculum regarding the effects of birth method on newborn health, the importance of breast milk, and the vaginal health-infection relationship. When comparing the participants’ pre- and post-test knowledge scores, a statistically significant difference was found.

The microbiota education provided has been linked to the success of the students who participated in the training, suggesting that it could serve as a model for microbiota awareness programs using the peer education approach. Since the results are limited to the students who took part in the training, expanding the program to a larger audience might help raise broader awareness about the importance of microbiota.

## CONCLUSION

The microbiota training provided raised awareness among midwifery students about the importance of microbiota and helped them establish the relationship between microbiota and newborn health, women's health, and diseases.

## Data Availability

Data used and analyzed in the current study are available from the corresponding author upon reasonable request.
